# Supplementary material for: A Global Screen for Assembly State Changes of the Mitotic Proteome by SEC-SWATH-MS
Source: Cell Syst. 2020 Feb 26;10(2):133–155.e6. doi: 10.1016/j.cels.2020.01.001 (PMC7042714; doi:10.1016/j.cels.2020.01.001)

Q93074 | MED12_HUMAN | MED12 ARC240 CAGH45 HOPA KIAA0192 TNRC11 TRAP230
Monomer MW [kDa]: 243.081 Monomer expected elution fraction: 32

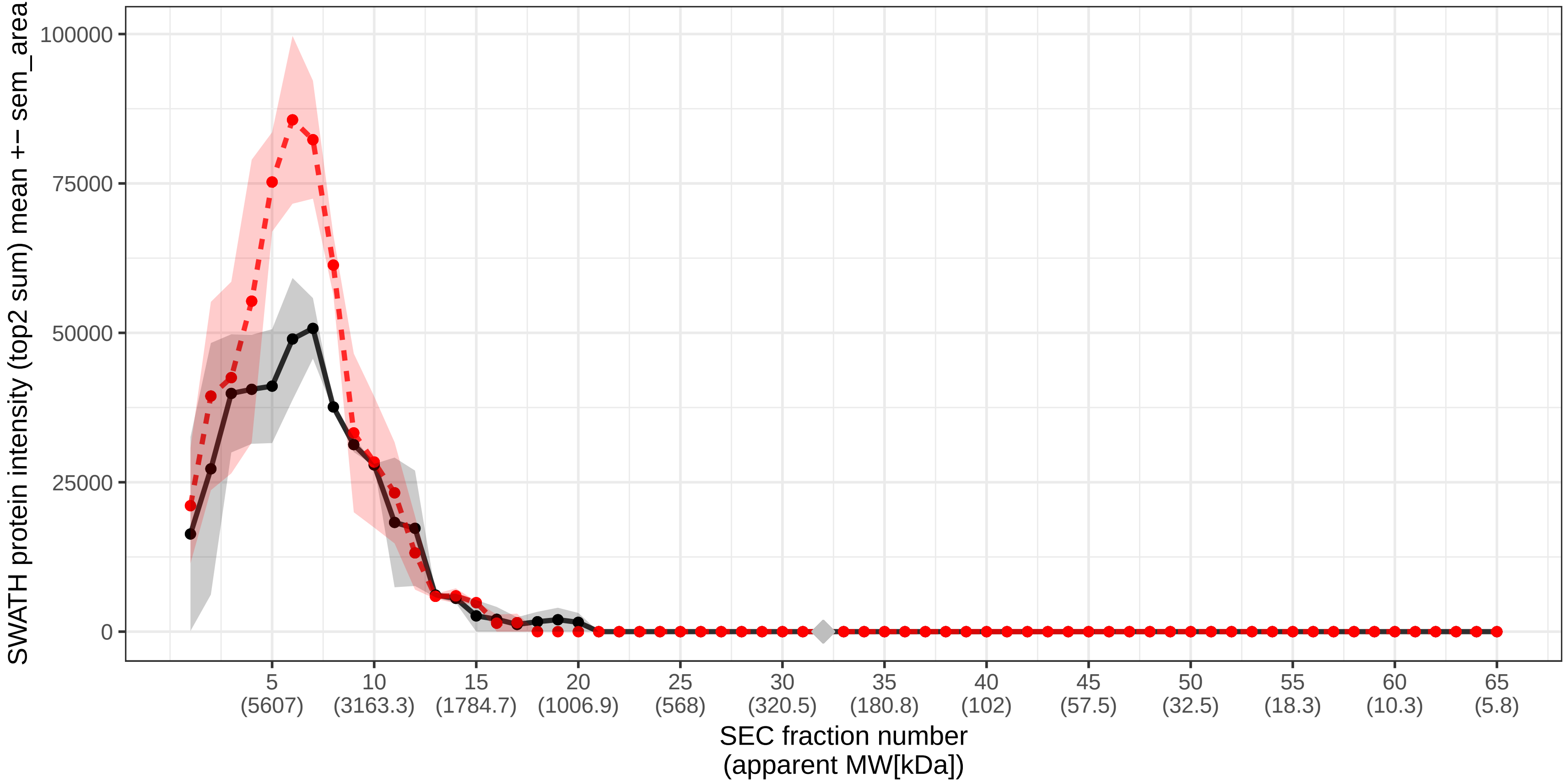

Supplement: Data S1. SEC-SWATH-MS Protein Chromatograms, Related to Figure 1 [file mmc6.zip › SECchrom_Q93074_MED12_HUMAN_MED12_ARC240_CAGH45_HOPA_KIAA01.pdf]
